# Biliary drainage combined with simultaneous ^125^I seed strand brachytherapy for the treatment of hilar cholangiocarcinoma

**DOI:** 10.1186/s12885-023-10868-5

**Published:** 2023-05-09

**Authors:** Chengzhi Zhang, Mengyao Song, Zhanguo Sun, Yi Fang, Yiming Liu, Kaihao Xu, Xinwei Han, Dechao Jiao

**Affiliations:** Department of Interventional Radiology, The First Affiliated Hospital of Zhengzhou University, 1 Jianshe East Road, Zhengzhou, 450052 China

**Keywords:** Hilar cholangiocarcinoma, ^125^I seed brachytherapy, Biliary drainage, Malignant obstruction

## Abstract

**Background:**

To evaluate the clinical efficacy of percutaneous biliary drainage (PBD) combined with ^125^I seed strand brachytherapy (ISSB) for the treatment of hilar cholangiocarcinoma (HCCA).

**Methods:**

The clinical data of 64 patients with HCCA (median age 62.5, male 29, female 35) treated in our department from April 2017 to April 2021 were retrospectively analyzed. Thirty-four patients in the experimental group (EG) were treated with PBD combined with ISSB, while 30 patients in the control group (CG) were treated with PBD alone. The primary study endpoints were technical success, clinical success and the 2-month local tumor control (LTC) rate. Secondary endpoints were early/late complications, median progression-free survival (mPFS) and overall survival (mOS).

**Results:**

The technical and clinical success in the EG and CG showed no significant differences (100 vs. 100%, 94.1 vs. 93.3%, *P* > 0.05). Both early and late complications showed no significant differences between the two groups (*P* > 0.05). The 2-month LTC rates were significantly better in the EG versus the CG (94.1% vs. 26.7%, 157.7 ± 115.3 vs. 478.1 ± 235.3 U/ml), respectively (*P* < 0.05). The mPFS and mOS were 4.3 (95% CI 3.9–4.7) months and 2.8 (95% CI 2.5–3.1) months and 13.5 (95% CI 10.7–16.3) months and 8.8 (95% CI 7.8–9.8) months, respectively, with significant differences (*P* < 0.05).

**Conclusion:**

PBD combined with ISSB is a safe and effective treatment for HCCA that can inhibit local tumors and prolong PFS and OS.

## Introduction

Hilar cholangiocarcinoma (HCCA) is a group of heterogeneous malignant tumors, accounting for 50% of extraphepatic cholangiocarcinomas [1]. HCCA often has no specific clinical manifestations before the bile duct is completely blocked, which means it rarely attracts the attention of patients and clinicians. The early symptoms of HCCA are mostly nonspecific symptoms, such as anorexia, decreased appetite, greasy stool, dyspepsia and epigastric discomfort, and some patients may have repeated bile duct infections [2]. Hyperbilirubinemia becomes noticeable with HCCA progression, but unfortunately, more than 70% of patients cannot undergo surgical resection because of its special location, invasive growth and close relationship with the blood vessels of the hepatic portal. Moreover, the recurrence rate and 5-year survival rate are 50–70% and 10–40%, respectively, even after R0 resection [3, 4], and there is still no definite, effective adjuvant treatment plan.

Radioactive ^125^I seed brachytherapy (RISB) is a minimally invasive treatment under modern imaging technology, and ^125^I seeds are permanently or temporarily implanted into the tumor, where they continuously release γ-rays to play a sustained killing role against the tumor [5]. There are two forms of RISB. One is that ^125^I seeds are permanently implanted into solid tumors by percutaneous puncture, and the other is that ^125^I seeds are placed within a catheter to form regular and linear ^125^I seed strands (ISSs), which are placed within a human lumen, such as the biliary duct [6] or blood vessels [7], by catheter and guidewire technology. RISB has the advantages of sustained low-dose radiation, high local dose accumulation, simple operation, easy protection, strong repeatability, low price and so on.

Inspired by high-dose rate ^192^Ir brachytherapy (HDRB) [8], RISB had been applied to extrahepatic middle and distal malignant biliary obstruction at our center before, aiming at prolonged stent patency (brachytherapy vs. control group: 368.0 vs. 220 days, *P* < 0.05) [9]. Due to the challenging anatomical position, few studies have focused on RISB in HCCA. Percutaneous biliary drainage (PBD) has been applied as the preferred strategy for hilar biliary obstruction in our clinical practice [10].

Our team developed a strategy where a drainage catheter was placed across the hilar obstruction to drain the bile and ISS was used to play an antitumor role. After 2 months of RISB, if the bile duct was unobstructed, the drainage catheter was removed; if it was not unobstructed, a stent was placed to open the obstruction, and then systemic chemotherapy was performed to enhance the antitumor effect. The present study describes our experience with this combination of treatment.

## Materials and methods

### General information

**Fig. 1:**
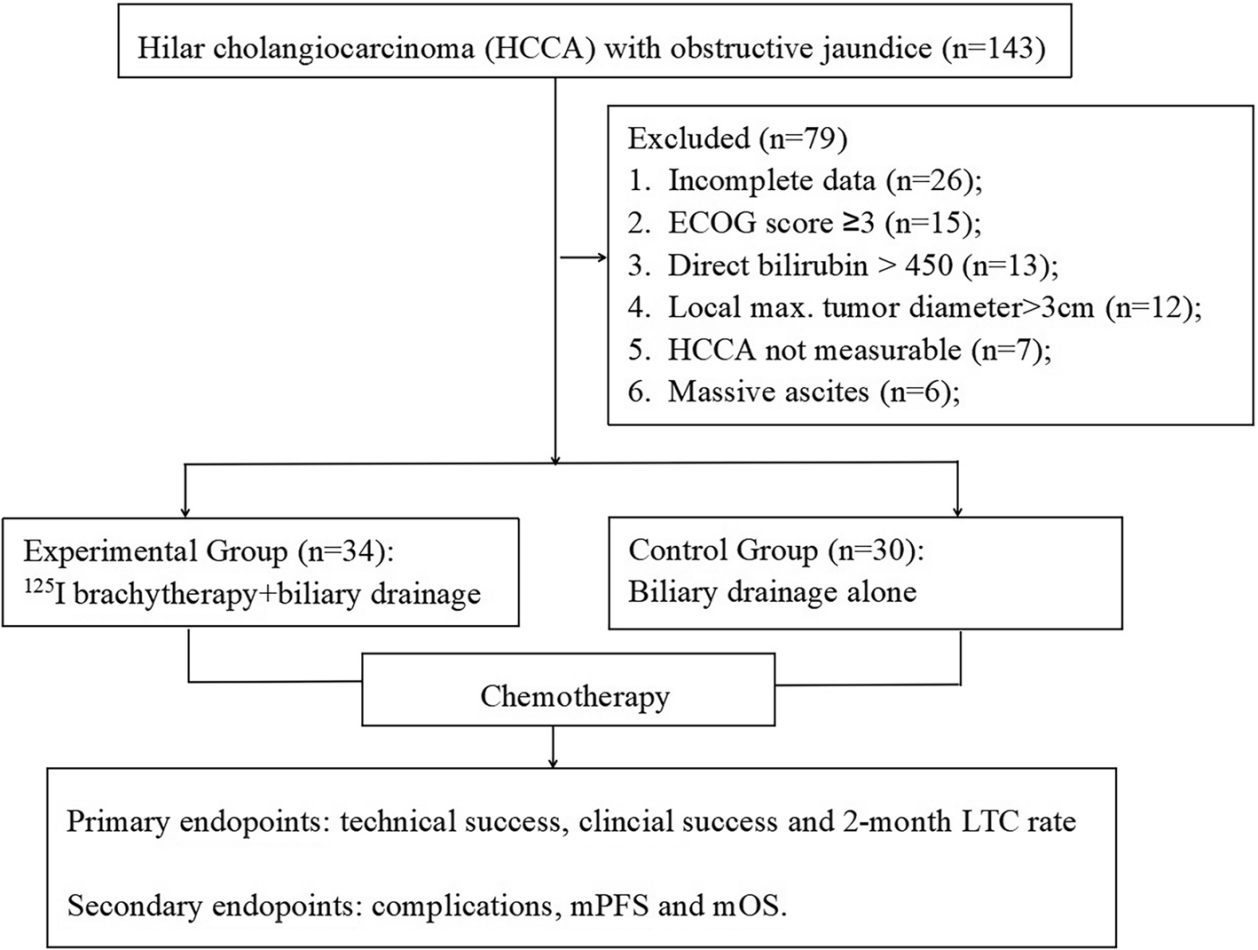
The study workflow

The Ethics Committees of the First Affiliated Hospital of Zhengzhou University agreed to this retrospective study. The clinical data of 64 patients with measurable HCCA (median age 62.5, male 29, female 35) in our department from April 2017 to April 2021 were retrospectively analyzed. Thirty-four patients in the experimental group (EG) were treated with PBD combined with ISSB from December 2018 to April 2021, while 30 patients in the control group (CG) were treated with PBD alone before December 2018. The inclusion criteria were as follows: (1) age 18–80 years; (2) clear evidence of malignant histology/cytopathology; (3) measurable lesions on imaging examination; (4) Eastern Cooperative Oncology Group (ECOG) score ≤ 2; (5) moderate to severe biliary dilatation; (6) existence of an appropriate puncture path on imaging examination; (7) refusal or inability to undergo surgery; and (8) no external radiotherapy before. The exclusion criteria were as follows: (1) benign biliary obstruction; (2) uncontrollable intractable ascites; (3) ECOG score ≥ 3; (4) severe coagulation disorder, such as platelet count ≤ 30 × 10^9^/L and prothrombin time > 25 s; (6) severe cardiopulmonary dysfunction; and (7) uncontrolled severe infection. Detailed information and the workflow are listed in Table 1 and Fig. 1, respectively.

**Table 1:** Baseline characteristics

Parameter	Experimental Group (*n* = 34)	Control group (*n* = 30)	*P* value
Age(year)	62.7 ± 10.9	61.5 ± 8.5	0.63
Sex(male/Female)	14/20	15/15	0.62
Tumor differentiation (Medium and low/highly)	9/25	8/22	0.99
Max Diameter (mm)	16.6 ± 6.7	17.6 ± 5.5	0.53
Obstruction length (mm)	48.5 ± 10.7	52.2 ± 8.0	0.13
Bismuth type (I/II/III/IV)	3/6/18/7	4/5/17/4	0.84
ECOG score (0/1/2)	8/8/18	5/10/15	0.63
Distant metastasis(Yes/no)	13/21	15/15	0.34
Biochemical index			
White blood cell (× 10^9^/L)	6.4 ± 1.6	6.5 ± 1.6	0.76
Platelet(× 10^9^/L)	175.1 ± 29.7	168.0 ± 24.1	0.30
Hemoglobin(g/L)	126.2 ± 16.5	121.7 ± 12.2	0.22
Albumin (g/L)	39.1 ± 2.7	38.7 ± 2.3	0.57
Glutamic pyruvic transaminase (U/L)	88.2 ± 35.8	97.0 ± 40.0	0.36
Glutamic oxaloacetic transaminase (U/L)	85.0 ± 36.5	98.3 ± 41.0	0.18
Total bilirubin (µmol/L)	168.1 ± 53.0	184.6 ± 24.1	0.22
Direct bilirubin (µmol/L)	131.5 ± 46.0	139.2 ± 42.8	0.50
Prothrombin time(s)	17.9 ± 2.9	18.0 ± 2.7	0.90
Serum CA19-9 (U/ml)	592.2 ± 253.8	507.4 ± 245.3	0.18
Follow up Chemotherapy (1–3/4–6 courses)	23/11	24/6	0.26

*PC* Pancreatic cancers, *GC* Gastric cancers, *LC* Liver cancers, *Second C* Chemotherapy, *TT* Target therapy, *ST* symptomatic treatments

### ^125^I seed strands (ISS)

**Fig. 2:**
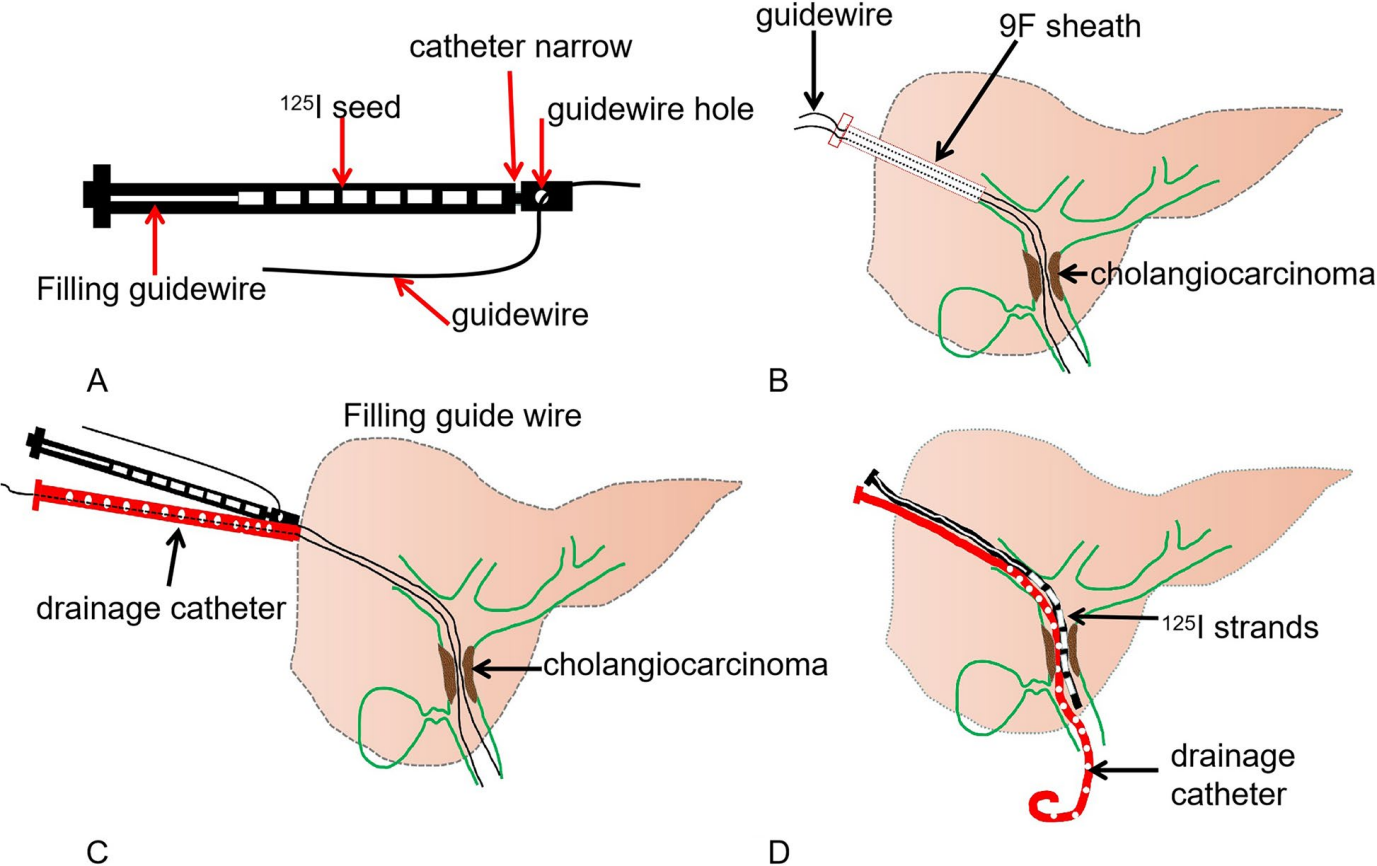
Schematic diagram of ^125^I seed strand (ISS) production and release process. A narrow catheter port was placed 2 cm away from the head end of the 3F medical catheter by burring methods. A small guidewire hole was opened 1.5 cm away from the head end, which accommodates the passage of a 0.035 inch guide wire. The ^125^I seeds were arranged one by one behind the narrow catheter, and the non-^125^I seed-filled part was fixed with a 0.018 inch soft filling guidewire to prevent.^125^I seed displacement (**A**). Double guidewires were inserted into the bile duct through a 9F short sheath (**B**). The ISS and drainage catheter were advanced by two guidewires (**C**). The 8.5F drainage catheter and ISS were distributed in parallel across the tumor obstruction (**D**)

Single ^125^I seeds (Tianjin Saide Biopharmaceutical Co., Ltd., China) were Type-6711 and cylindrical (0.8 mm × 4.5 mm) with titanium capsules emitting low-dose γ-rays (35.5 keV) and soft X-rays (28.6 keV). The radioactivity per seed was 0.80 mCi with a half-life of 59.6 days, and the radius of the effective antitumor activity was 17 mm. The ISS were prepared by: A narrow catheter was placed 2 cm away from the head end of a 3F medical catheter by the burring method (Cook, USA). A small hole was opened 1.5 cm away from the head end to accommodate passage of a 0.035 inch guide wire. The length of the ISS was determined by the biliary obstruction, ensuring that the ISS exceeded the obstruction by 2 cm, and the non-^125^I seed-filled part was fixed with a 0.018 inch soft filling guide wire to prevent ^125^I seed displacement (Fig. 2). The absorbed cumulative dose was verified by a treatment planning system (TPS, Beijing Tianhang Kelin Technology Development Co., Ltd.).

### Procedures (Biopsy-PBD-ISSB)

Routine blood tests, liver and kidney function, electrolytes, coagulation function, electrocardiography and abdominal enhanced CT/MR (including magnetic resonance cholangiopancreatography, MRCP) were performed 3–5 days before the operation.

**Fig. 3:**
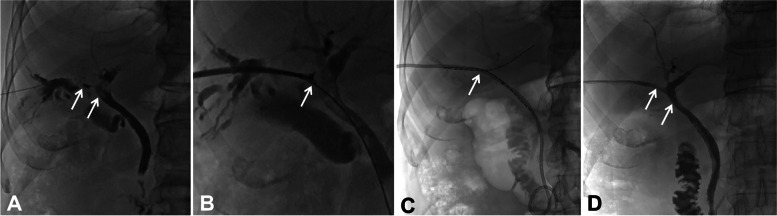
Woman, 55 years old. Percutaneous transhepatic cholangiography was performed to visualize the tumor location (arrow) and Bismuth type IV under fluoroscopy (**A**); percutaneous transhepatic forceps biopsy (arrow) was performed at the Hilar obstruction through a 9F sheath (**B**); biliary drainage and double ^125^I strands (arrow) were inserted across the Hilar cholangiocarcinoma (**C**); postoperative cholangiography showed that the tumor disappeared and there was biliary patency (arrow), and both the drainage catheter and.^I25^I strands could be directly removed (**D**)

Fifty milliliters of a mixture of deszocin (10 mg) and dexmedetomidine hydrochloride (400 µ g) was intravenously infused (4 ml/h) by a pump to obtain a satisfactory pain control state. The patient lay prone on the DSA examination table (Artis Zeego, Siemens, Germany). After local disinfection and draping, local anesthesia was performed with 2% lidocaine (5 ml), and the dilated biliary branch was successfully punctured by the Seldinger technique using a PTC puncture set (Cook, USA) under US and DSA guidance. Biliary cholangiography was performed to confirm the obstruction and length using a 5F KMP catheter (Cook) and a 0.035 inch guidewire (110 cm in length, Terumo, Japan), and the catheter was manipulated to open the obstruction and enter the duodenum with the help of a guidewire. A strength guidewire (260 cm long, Terumo, Japan) was exchanged, and a 9F sheath was introduced along the strength guidewire. The biopsy forceps (6 mm in diameter, Microtech. Nanjing Medical Co., Ltd) were introduced through the sheath for sampling (Fig. 3), and the 12-14F internal and external drainage tubes (Cook) were implanted after the biopsy. The lateral foramen should span the tumor-occluded segment and be able to drain bile smoothly. The puncture track was established when HCCA was confirmed by pathology 3–4 days later.

**Fig. 4:**
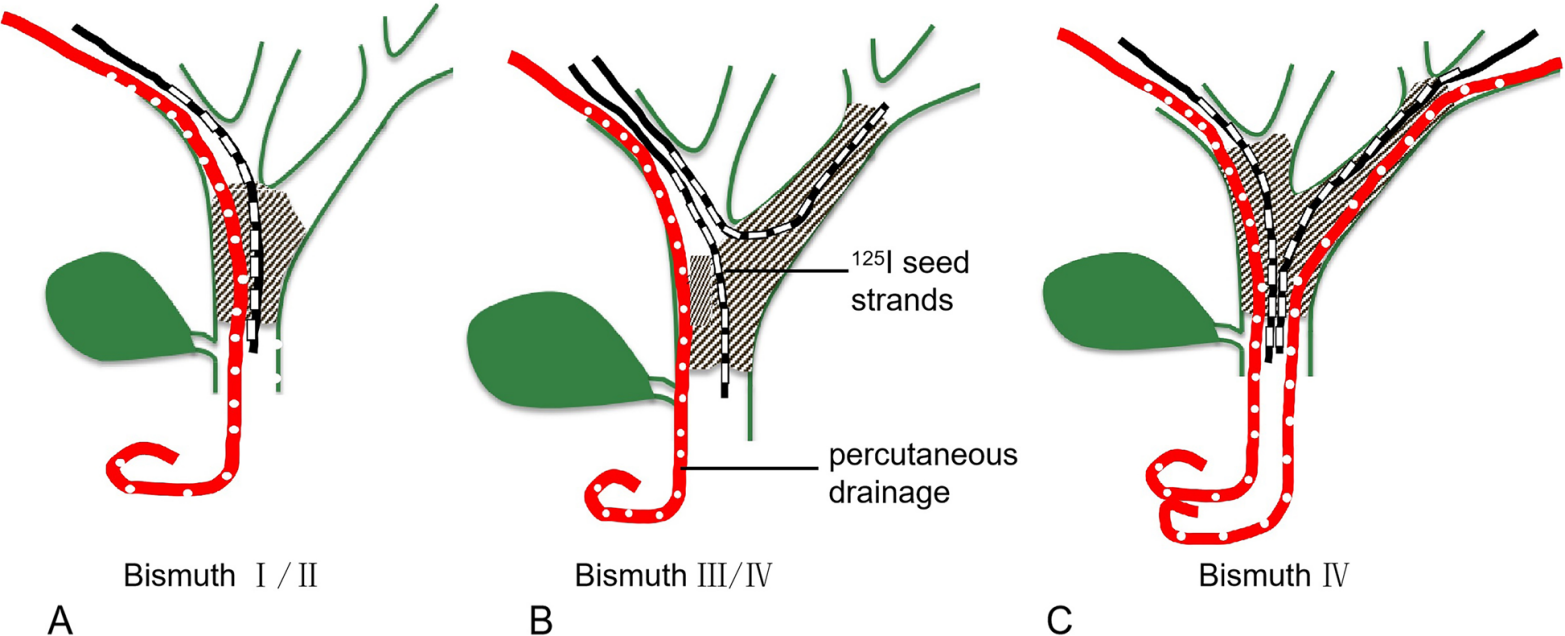
Schematic diagram of single or double ^125^I seed stands combined with percutaneous biliary drainage therapy for different Bismuth types

**Fig. 5:**
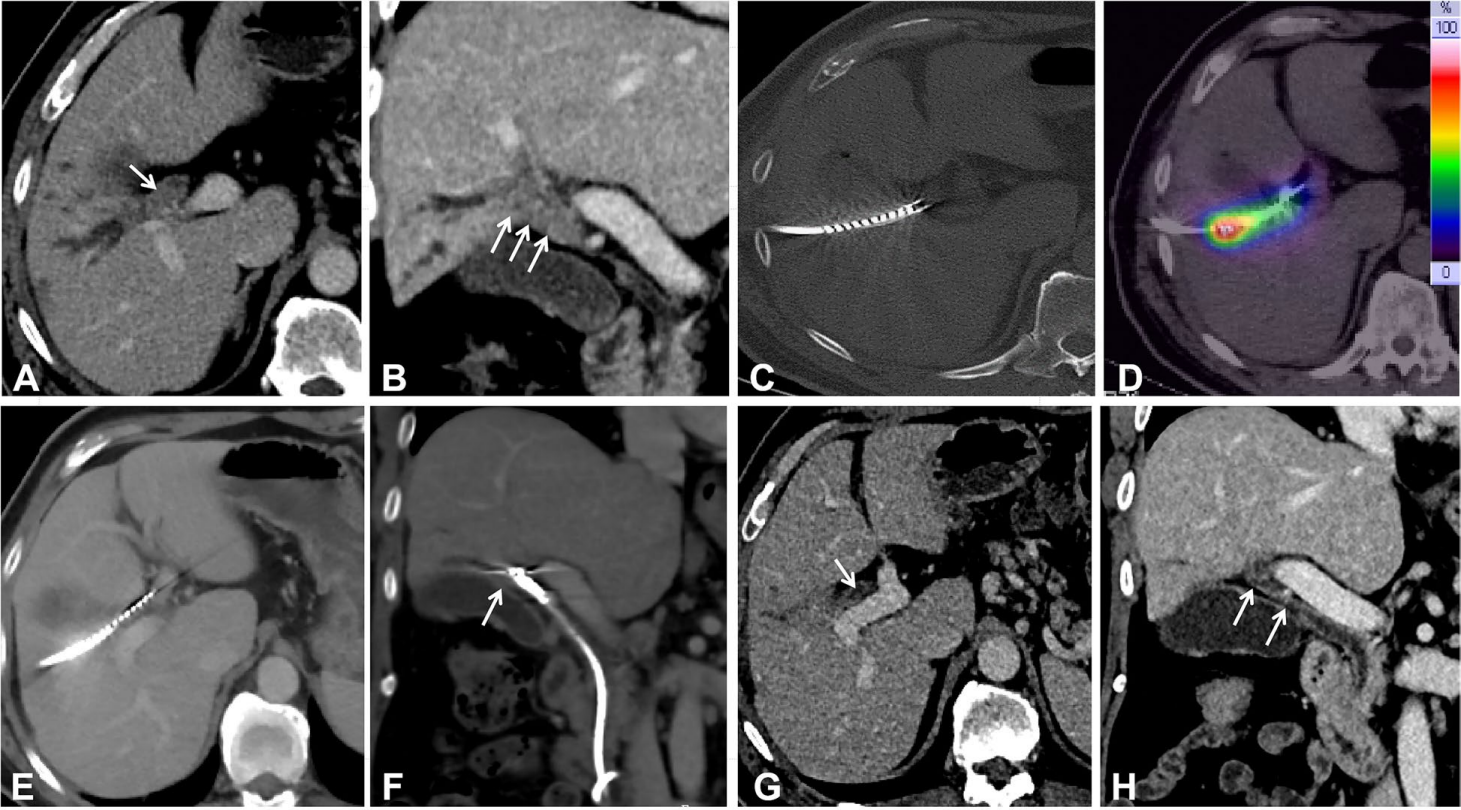
Woman, 55-year-old, pretreatment enhanced CT showed HCCA in the transverse (**A**) and coronal positions (**A**) (arrow); posttreatment CT showed that ISS was distributed along the biliary obstruction (**C**); γ-ray covered HCCA on SPECT with a high local dose and an extremely low dose to the normal surrounding tissues (**D**); posttreatment enhanced CT showed that HCCA disappeared in the transverse (**E**) and coronal positions (**F**) (arrow), and ISS could be successfully pulled out without biliary stenting. After RISB, the patient completed 4 courses of GP chemotherapy, and the local tumor was evaluated as a complete response after 1 year on enhanced CT (**G**, **H**)

The 9F short sheath (23 cm length, Cook) was arranged along the puncture track, triple guidewires were advanced to the bile duct, one guidewire entered into the contralateral common bile duct, and the remaining two guidewires entered into the common bile duct and duodenum. A 6 × 60 mm balloon was used to dilate the obstruction. Only the triple guidewires were retained after 9F sheath and balloon withdrawal. 3F ISSs were advanced by two guidewires using a rapid exchange technique (Fig. 4) according to the biliary obstruction. An 8.5 F biliary drainage catheter (45 cm in length, Cook) was advanced along the skin-bile duct-duodenum guidewire, and the lateral foramen spanned the tumor-occluded segment to smoothly drain the bile. The external-internal drainage catheter and both ISSs were fixed together to prevent displacement. If the left and right bile ducts were not completely blocked, the single approach was used, while both approaches were used if the left and right branches were mutually blocked. The Y-shaped particle strips were implanted through the left and right approaches (Fig. 5).

### Follow-up

**Fig. 6:**
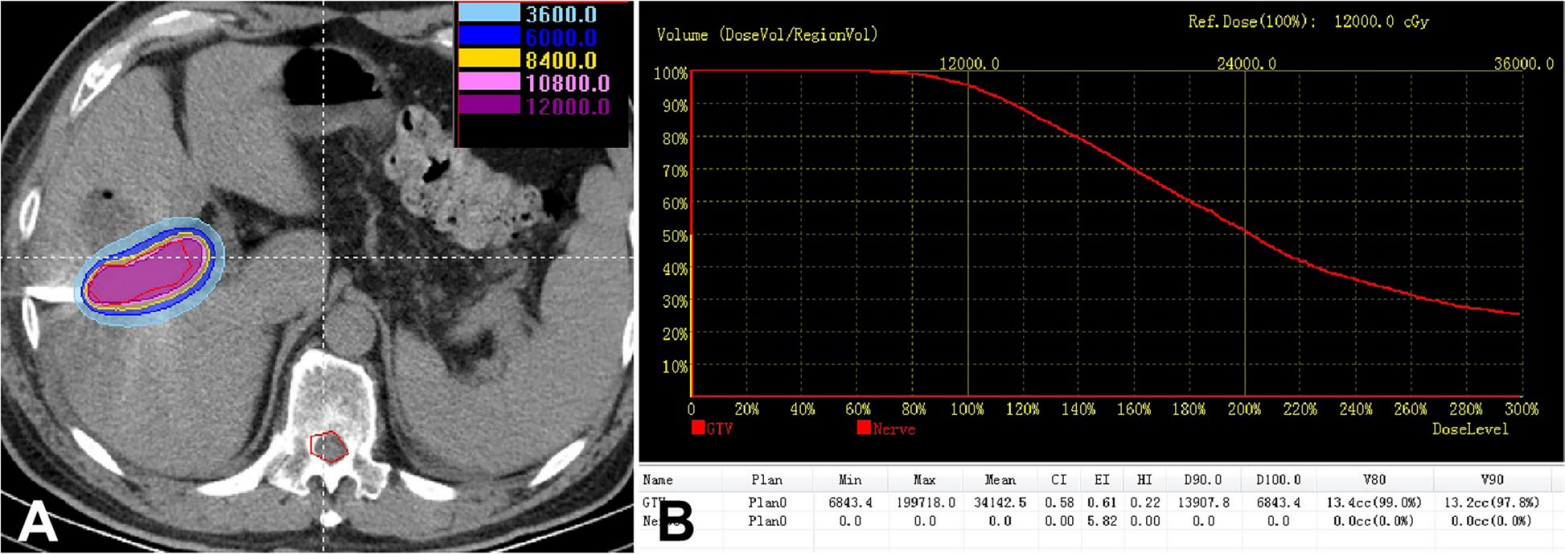
Evaluation of irradiation dose after treatment by the treatment planning system (TPS). Different colors represent different dose ranges (**A**); dose volume histogram of the tumor and organs at risk on the TPS (**B**)

Local SPECT was performed within 3 days after the operation to observe the γ-ray coverage, and the local cumulative dose was calculated by the TPS (Fig. 6). The organs at risk (OAR) was set as the spinal cord at the same level. Routine blood examination, liver and kidney function, electrolytes and upper abdominal enhanced CT were performed to understand the biochemical index and local tumor control (LTC) at the 2 month (one half-life of the ^125^I seed) follow-up. If the tumor shrank and the bile duct was unobstructed (more than 50% of the bile duct diameter), the ISS and biliary drainage catheter were removed. If there was still stenosis (whether it was neoplastic or radioactive cicatricial hyperplasia), the ISS was removed, and biliary stenting or PBC was performed. All cases were recommended to receive chemotherapy (gemcitabine plus cisplatin) after their bilirubin decreased to 50 µmol/L and their Karnofsky score was more than 70 after evaluation by a hospital multidisciplinary consultation.

### Definition

Technical success was defined as successful PBD and ISS placement. Clinical success was defined as bilirubin decreased by 20% within 1 week. According to the operation guidelines of the International Society of Interventional Radiology (SIR), complications are divided into early (≤ 30 days) and late (> 30 days) complications [11]. The local tumor response of ISS to HCCA were evaluated by the response evaluation criteria in solid tumors (RECIST), including complete response (CR), partial response (PR), stable disease (SD) and progressive disease (PD). CR was complete disappearance of local tumor; PR was that maximum transverse tumor diameter decreased by 30%; SD was maximum transverse tumor diameter decrease between PR and PD, PD was maximum transverse tumor diameter enlarged by 20%.

Local control rate (LCR) = CR + PR + SD/Total patients number × 100%. Progression-free survival (PFS) was defined as the time from our hospital's diagnosis to the patient's tumor progression or death. Overall survival (OS) was defined as the time from the diagnosis to the end of the follow-up or the patient's death.

### Statistical analysis

SPSS 22.0 software (Version 21.0, Chicago, Illinois) was used for statistical analysis. All data are expressed as numbers (mean ± standard deviation), percentages and ranges. All parameters between both groups were compared by an independent samples t-test, χ^2^ test or *Fisher's* exact test. The Mann‒Whitney test was used to compare the difference in the variance (difference between pro- and postprocedure). The Kaplan‒Meier test was used for the PFS and OS analyses. *P* < 0.05 was considered statistically significant.

## Results

The clinical data of 64 patients with HCCA (median age 62.5, male 29, female 35) in our department from April 2017 to April 2021 were retrospectively analyzed. Their baseline characteristics were well balanced and are listed in Table 1. The first-line chemotherapy regimen for both groups was gemcitabine combined with cisplatin. These included gemcitabine at doses 850–1000 mg/m^2^ intravenously on day-1 and 8 and cisplatin at doses 40 mg/m^2^ intravenously on days-2 and 3. The treatment cycle was 3 weeks. The second-line treatment was albumin paclitaxel combined with Teglio capsules. The albumin paclitaxel were used on day-1 and 8 (dose: 125 mg/m^2^). The technical success was 100% in all cases. Bilirubin in two cases decreased by less than 20% within 1 week in both groups (4 cases were Bismuth type IV with a small amount of biliary bleeding after PBD), which means clinical success was 94.1 and 93.3% in the EG and CG, respectively, without significance (*P* > 0.05). A total of 1156 seeds were used, with 26 single and 38 double ISS. The mean ^125^I seed number was 34.0 ± 6.0 (range: 20–44) in the EG, and the D90 and OAR doses calculated by the TPS were approximately 80.6 ± 13.8 Gy (range 54.1–113.7 Gy) and (1.8 ± 0.4) Gy, respectively.

After 2 months of ISSB, the ISS could be removed in only 7 cases (20.6%) with satisfactory biliary angiography, and 27 cases underwent biliary stenting (*n* = 14) or PBD (*n* = 13) in the EG group (Table 2). Nineteen and 11 patients underwent biliary stenting or continuous PBD in the CG group after 2 months of biliary drainage.

**Table 2:** Intra- and post operative parameters

Parameter	Experimental Group (*n* = 34)	Control group (*n* = 30)	*P* value
Technical success (%)	100%	100%	-
Clinical success (%)	94.1%	93.3%	0.90
Procedure time (min)	42.3 ± 5.3	40.1 ± 7.5	0.18
Approach (Left/right)	7/27	5/25	0.67
Biochemical indexat 2-month			
White blood cell (× 10^9^/L)	5.5 ± 1.3	5.6 ± 1.3	0.78
Platelet(× 10^9^/L)	173.2 ± 27.8	166.1 ± 26.0	0.30
Hemoglobin(g/L)	124.8 ± 15.0	122.0 ± 12.2	0.41
Albumin (g/L)	39.9 ± 2.5	39.6 ± 1.6	0.61
Glutamic pyruvic transaminase (U/L)	48.4 ± 13.7	43.4 ± 12.1	0.13
Glutamic oxaloacetic transaminase (U/L)	50.5 ± 11.7	48.0 ± 8.9	0.35
Total bilirubin (µmol/L)	39.2 ± 6.6	38.1 ± 5.1	0.45
Direct bilirubin (µmol/L)	24.1 ± 7.0	23.8 ± 4.8	0.89
Prothrombin time(s)	17.9 ± 2.9	18.0 ± 2.7	0.90
Max. Diameter (mm)	6.6 ± 5.2	23.2 ± 6.2	0.00
LTC rate (CR + PR + SD/total × 100%)	94.1%	26.7%	0.00
Bile duct patency (Yes/no)	7/27	0/30	0.00
Following biliary stenting/catheter drainage (yes/no)	14/13	19/11	0.38
mPFS(month, 95%CI)	4.3 (95%CI 3.9–4.7)	2.8 (98%CI 2.5–3.1)	0.00
Median overall survival (month, 95%CI)	13.5 (95%CI 10.7–16.3)	8.8 (7.8–9.8)	0.00

*mPFS* Median progression free survival, *mOS* Median overall survival, *LTC* Local tumor control, *CR* complete response, *PR* Partial response, *SD* Stable disease

At the 2-month follow-up, the maximum diameter decreased from 16.6 ± 6.7 mm to 6.6 ± 5.2 mm in the EG (*P* < 0.05), while the diameter increased from 17.6 ± 5.5 mm to 23.2 ± 6.2 mm in the CG (*P* < 0.05).

Regarding early complications, 4 (11.8%) and 3 (10.0%) cases in the EG and CG, respectively, had biliary infection (*P* > 0.05), with fever (≥ 38.5°) with chills, which was resolved by antibiotics and acid inhibition for 3–7 days. The others were minor complications, which showed no significant difference between the groups (*P* > 0.05). No severe complications, such as massive bleeding, liver abscess, or fistula formation, occurred (Table 3).

**Table 3:** Complications at both groups

Parameter	Experimental Group (*n* = 34)	Control group (*n* = 30)	*P* value
Early complications			-
Skin bile leakage	8	3	0.14
Cholangitis	6	4	0.64
Self limited hemobilia	9	4	0.19
Biliary infection	4	3	0.82
Late complications			
Skin bile leakage	11	4	0.12
Cholangitis	4	5	0.57
Cholecystitis	2	2	0.85
^125^I seed strands migration	2	0	0.11

**Fig. 7:**
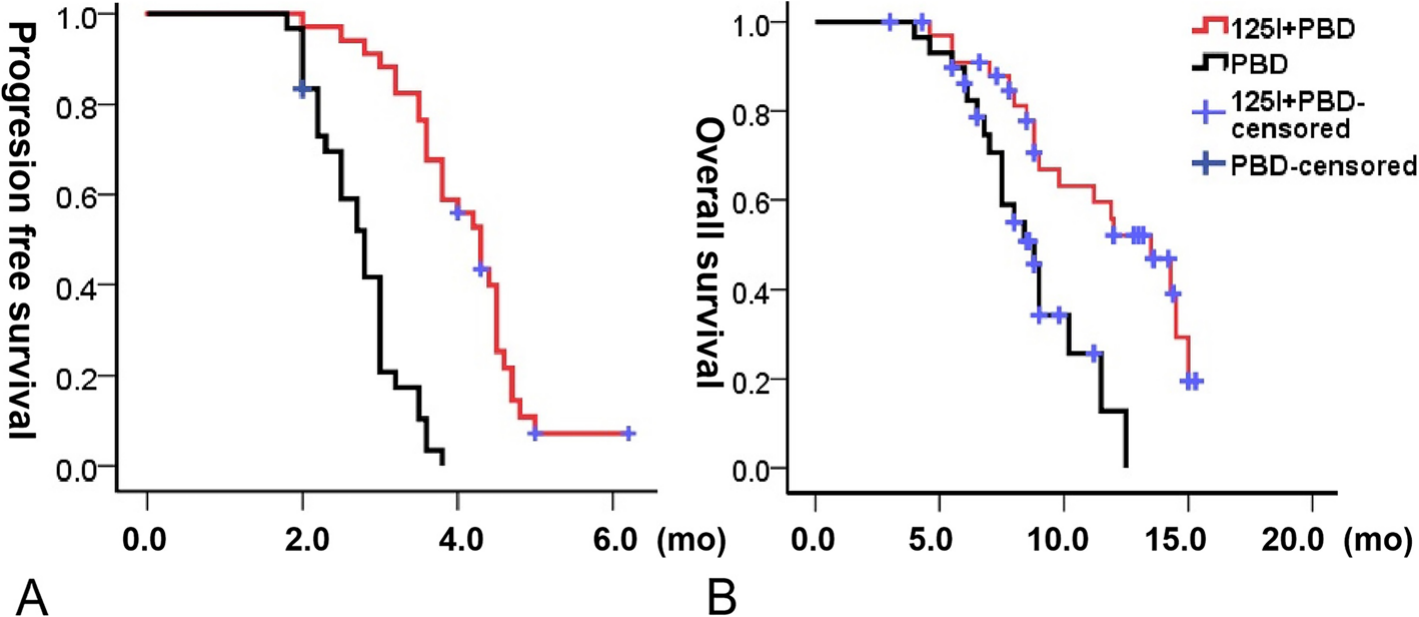
Progression-free survival and overall survival in both groups

**Fig. 8:**
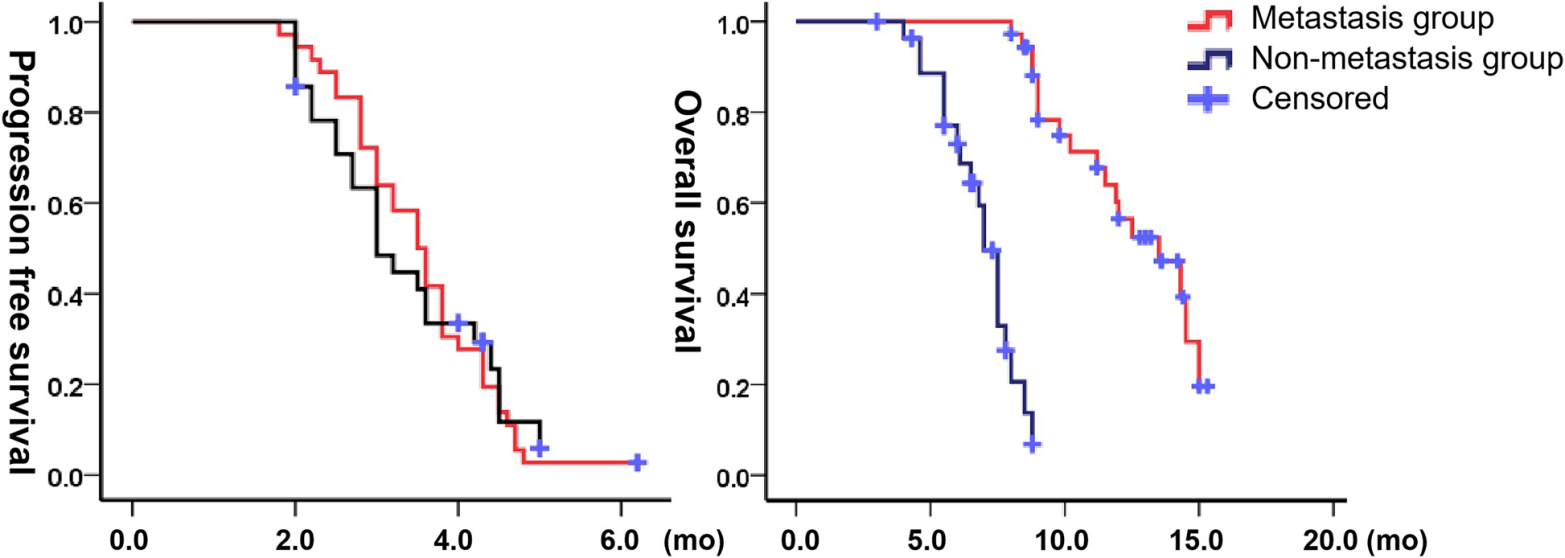
Progression-free survival and overall survival in metastasis and non-metastasis groups

During the mean follow-up of 9.2 months, the mPFS and mOS were 4.3 (95% CI 3.9–4.7) months and 2.8 (98% CI 2.5–3.1) months and 13.5 (95% CI 10.7–16.3) months and 8.8 (7.8–9.8) months, respectively, with no significant differences (*P* > 0.05). Thirty-seven patients died, 18 and 19 patients in the EG and CG, respectively. The reasons for death were progression of disease (*n* = 28), multiple organ failure (*n* = 7), lung infection (*n* = 1), and pulmonary embolism (*n* = 1). The 6-/12-month OS rates were 90.9/52.1% in the EG and 86.1/12.9% in the CG (Fig. 7). Because some of the patients in the group had metastasis before ISS, metastasis and non-metastasis groups were advanced analyzed. The mPFS were 3.0 (95%CI 2.5–3.5) months and 3.5 (95%CI 3.1–3.9) months at metastasis and non-metastasis groups respectively, which showed no significance (*P* = 0.98). The mOS were 7.0 (95%CI 6.5–7.6) months and 13.5 (95%CI 10.9–16.1) months at metastasis and non-metastasis groups respectively, which showed no significance (*P* = 0.00) (Fig. 8).

## Discussion

HCCA is a malignant tumor that occurs at the junction of the left and right bile ducts and was first described by Klatskin in 1965 [12]. Hyperbilirubinemia resulting from malignant biliary obstruction in these patients is prone to causing liver failure, and decreasing biliary pressure by PBD or stenting can rapidly relieve the jaundice, which lays a foundation for further antitumor treatment [13]. Therefore, all clinicians should focus on how to quickly relieve the symptoms and reduce the tumor burden simultaneously. Liver transplantation can undoubtedly achieve long-term results in selective cases, but the high costs, the shortage of liver resources and the long-term use of antirejection drugs limit the wide application of this strategy [14]. Radical resection (i.e., R0 resection) is another hope for long-term survival for patients with HCCA, but the incidence of postoperative complications is as high as 14% ~ 36% [4, 15], mainly due to insufficient residual liver volume and bile fistula. Although portal vein embolization [16], 3D printing [17] and biliary reconstruction technologies [18] have been applied to help achieve R0 resection, the short-term recurrence was reported to be more than 50%, especially at the anastomosis and liver, and the 5-year OS is less than 20% [19].

For those who cannot undergo or refuse surgery, many treatments have been tried, such as external beam radiotherapy (EBRT) [20], photodynamic therapy (PDT) [21] and HDRB [22]. Unfortunately, all of them had subjective and objective shortcomings. EBRT may be influenced by limited dose tolerance of the surrounding organs (liver, stomach, duodenum), unclear delineation of the HCCA and respiration interference. PDT is limited by its expensive photosensitizers, optical fibers and postoperative skin photosensitivity. HDRB is not common in China because of the expensive equipment, high standards of protection, and complexity of the operation.

RISB is a typical conformal brachytherapy that emits continuous low-dose γ-rays within 1.7 cm, which have little effect on surrounding normal tissues. It has achieved remarkable efficacy in the control of solid tumors, such as prostate cancer [23], lung cancer [24], pancreatic cancer [25] and metastatic tumors [26]. ISS represents a feasible and effective therapy with 100% technical success without severe complications in this study. In fact, ISS served as an ideal treatment with its own advantages: (1) ISS is suitable for the curved structure of the bile duct, which can maximize the proximity to the tumor and play to the advantages of brachytherapy; (2) the irradiation dose accepted by the surrounding normal tissues is very low during RISB, and the OAR (spinal cord) received only 1.8 Gy in this study; (3) ISS delivering sustained irradiation can oxygenize hypoxic cells, and then the tumor cells will be more sensitive to irradiation; (4) ISS placement can be performed under local anesthesia during PBD, and the low cost (average cost 2500 dollars in the study) can be accepted by the majority of low-income families; (5) RISB is easy to undergo, and patients can enjoy warm care from their families at home, not in the hospital; and (6) the ISS can be withdrawn immediately when complications occur or the RISB is completed.

Since Bismuth type III/IV are multiple biliary branch obstructions, the PBD needs 4–6 weeks to recover to a satisfactory level [27], during which there is no antitumor treatment. This study makes full use of the physical characteristics of ^125^I, which can realize the double functions of bile drainage and brachytherapy, serving as a suitable bridge to additional systemic treatment for unresectable HCCA. The 2 month LTC rate (94.1% vs. 26.7%), mPFS (4.3 vs. 2.8 months) and OS (13.5 vs. 8.8 months) increased by 252, 53 and 53%, respectively, in the EG. Considering that the OS of HDRB [28] and chemotherapy (platinum based) [29] were 9.2 and 7.3 months in a previous study, respectively, the current results are satisfactory. Isocitrate dehydrogenase isoenzyme 1 (IDH1) mutations have a high occurrence in recent studies, and ivosedinib has been developed as a small molecule inhibitor of mutant IDH1 (mIDH1) [30]. Biliary forceps biopsy can be used to detect IDH1 mutations, and RISB combined with ivosedinib may be a good research direction in the future. Considering the importance of the tumor staging, the sub-groups of metastasis and non-metastasis were analyzed further. The results showed that there was no difference in PFS but significant difference in OS, which further indicated that ISS was still a local treatment, After all, malignant tumor was still a systemic disease. ISS for localized HCCA may benefit more, but the objective impact still needed to be further increased in sample size and extended observation events.

Liu et al. [31] first reported a pilot study of RISB combined with stenting in 11 cases with malignant biliary obstruction in 2009. Chen et al. demonstrated the safety of RISB in animals [32] and in a small sample clinical study [33]. The ISS was arranged outside the biliary stent to increase stent patency. From then on, an increasing number of hospitals have reported their positive experiences using single or double ISS to increase stent patency. The studies produced in the last 5 years are listed in Table 4, and a new type of interventional device has been developed to facilitate a more standardized and unified treatment scheme. These studies showed a technical success of 100%, complications 2.2–39.4%, stent patency 6.4–12.9 months and OS 6.4–12 months [34–43]. The D90 was 80.6 Gy, which cannot be achieved by EBRT (45–50 Gy delivered at 1.8–2 Gy/fraction) or HDRB (30 Gy delivered at 5 Gy/fraction) [44], which may explain the high LTC. Until now, no specialized TPS has been developed for ISS, and this is worth further study.

**Table 4:** Clinical studies on ^125^I strands brachytherapy on malignant biliary obstruction within recent 5 years

Year/study/Patients	Treatment design	Technical success	Cumulative dose	complications	Outcomes
2022/Rs/42 [35]	EG: stent + double ISSB CG: stent	100 vs 100%	125 and 86 Gy at 0.5 and 1 cm from ^125^I stands	Minor complications (27.2 vs 25%, *p* > 0.05)	Tumor shrinkage (0.89 vs -1.19 cm); mSP ( 8.76 vs 3.55 mo); mOS (252.5 vs133.5 day), all *P* value *p* < 0.05
2022/Rs/48 [36]	EG: double ISSB + BD + CT CG:BD + CT	100 vs 100%	D90: 83.5 Gy	Early complication (12.5 vs 16.7%, *p* > 0.05)	mSP (7.8 vs 5.7 mo, *p* < 0.05); mOS( 9.4 vs 8.2 mo, *p* > 0.05)
2021/Rs/13 [37]	SAR: Intraluminal RFA + single ISSB	100%	27.2 Gy at 0.5 cm from ^125^I stands	Minor complications 23.1%	mSP (239 days, 95%CI:187–291) mOS (298 days, 95%CI:239–358)
2021/Rs/67 [38]	EG: stent + singel ISSB CG: stent	100 vs 100%	Not available	Not available	mSP(9.0 vs 6.0 mo, *p* < 0.05); mOS (11.0 vs 7.0 mo, *p* < 0.05)
2021/Rs/58 [39]	EG: BD + singel ISSB CG: BD	100 vs 100%	50–80 Gy at dose reference point	Grade3-4 complications (30% vs 39.4%, *p* > 0.05)	Median obstruction free time(7.0 vs 5.0 mo, *p* < 0.05); mOS (9.0 vs 6.0, *p* < 0.05)
2020/Rs/110 [40]	EG:stent + single ISSB CG:BD	100 vs 100%	Not available	Biliary infection (41.8% vs 18.2%, *p* < 0.05)	Clincial benefic rate (61.8% vs54.5% *p* < 0.05) mOS (13.4 vs 12.7, *p* < 0.05)
2020/Rs/84 [41]	EG:stent + single ISSB CG:BD	100 vs 100%	Not available	Complications (1.1 s 13.3%, *p* > 0.05)	mSP (231.6 vs 110.4 days, *p* < 0.05) mOS (310.6 vs 173.2 days, *p* < 0.05)
2020/Rs/76 [42]	EG:stent + single ISSB CG:stent	100 vs 100%	Not available	Complications (50.0 s 38.9%, *p* > 0.05)	mSP (387.0 vs 121.0 days, *p* < 0.05) mOS (177.0 vs 123.0 days, *p* > 0.05)
2019/Rs/184 [43]	EG:BD + single ISSB CG:BD	100 vs 100%	Not available	Minor complication (12.7 vs 13.3%, *p* > 0.05)	Biliary re-obstruction (9.5% vs 35.2%, *p* < 0.05) mOS (12.0 mo vs 9 mo, *p* > 0.05)
2019/Rs/132 [44]	EG:stent + single ISSB CG:stent	100 vs 100%	Not avaluable	Major complications (0 vs 2.2%, *p* > 0.05)	mSP (194.0 vs 86.0 days, *p* < 0.05) mOS (194.0 vs 96 days, *p* > 0.05)
			Not avaluable		

*Rs* Retrospective study, *EG* experimental group, *CG* Control group, *ISSB* 125I stands brachytherapy, *CT* Cheotherapy, *BD* Bliary drainange, *Mo* months, *D90* 90% of tumor volume receives radiation dose, *SAR* Single arm research, *mOS* median overall survival, *RFA* Radiofrequency ablation, *mSP* median Stent Patentcy

Regarding complications, 23.5 cases (13.5% higher than the CG) had skin bile leakage. The reason may be that the drainage catheter and ISS were placed through one puncture approach, and the patient's local skin was loose, which easily led to bile leakage to the skin, thus affecting the patient's quality of life. Therefore, future research and the development of integrated round drainage catheters would be more suitable for clinical application scenarios.

There are still some limitations to this study, such as the small sample size, short follow-up period, retrospective design, and bias of a single center. What’s more, PFS and OS depends on several factors including general condition, tumor factors, or sensitivity to treatments, the determined curative effect on both endopoints is still unknown, which needs further study. However, PBD combined with ISSB was safe and effective for treating HCCA, inhibiting the local tumor and prolonging PFS and OS. Prospective randomized controlled studies are needed in the future to confirm these findings.

## Data Availability

In addition to the raw data in the manuscript, the datasets used are available from the corresponding author on request.
